# Our academic heritage from CR Conti—A lifetime committed to education and excellence

**DOI:** 10.1002/clc.23912

**Published:** 2022-09-27

**Authors:** Carl J. Pepine

**Affiliations:** ^1^ Division of Cardiovascular Medicine University of Florida College of Medicine Gainesville Florida USA

**Keywords:** CR Conti, lifetime achievement, in memorium

## 1 INTRODUCTION

When Dr. Wenger asked me to write about the professional contributions made by CRC, on reflection I realized that this need not be about his CV, but more about the fascinating life that he enjoyed building our program at the University of Florida (UF) as an internationally prominent cardiologist and educator and the exhilaration of practicing with others with whom he was privileged to be involved. I thought, perhaps I could shed some light on the standards of excellence involved in being a cardiovascular disease (CVD) expert and how CRC emulated them.

At the time of his unexpected death, “Dick” was Emeritus Professor in Cardiovascular Medicine and an academician working at the UF in Gainesville. He had been involved during his working life evaluating and treating patients with known or suspect CVD and searching for evidence that one treatment is superior or at least not inferior to other approaches.

I trust that this piece will be interesting for many people, not only for other cardiologists. I have tried to portray key aspects of his professional life as a cardiologist in this article, starting from his career at UF in 1974, to after his retirement in 2014, to his death in 2022. I explain in a sequential way how his life as a cardiologist developed in a specific direction in to an internationally recognized academic cardiologist.

## 2 HIS INITIAL YEARS AT THE UF

Dick's arrival at the UF, along with the four new faculty that he recruited, including myself, provided a boost of enthusiasm, new ideas and strength in teaching, patient care and research. He became a favorite of our medical students, residents, and fellows, regularly lecturing on all clinical topics in CVD. In research he brought two NIH projects: National co‐operative randomized trial comparing surgical and medical therapy in unstable angina and an R01 evaluating the aortic blood flow velocity in myocardial ischemia.

## 3 ACUTE CORONARY SYNDROMES

Dick was very interested in acute manifestations of coronary artery disease (CAD). One of his earliest and important accomplishments was a leadership role in the NHLBI‐sponsored National Cooperative Study comparing intensive medical therapy (IMT) with urgent coronary bypass (coronary artery bypass graft [CABG]) surgery for acute management of unstable angina (1972–78). Beginning while he was at Johns Hopkins, he brought this pioneering multicenter prospective, randomized trial of IMT versus CABG to UF in 1974. The trial confirmed that such patients could be stabilized by IMT, which included propranolol and long‐acting nitrates in pharmacologic doses, with good control of angina in most cases and no increase in early mortality or myocardial infarction.^1^, ^2^, ^3^, ^4^ Later, if angina failed to respond to IMT, elective CABG could be performed with a lower risk and with good clinical results. The researchers also observed similar results for patients with proximal left anterior descending obstructive CAD, previously believed by many to be a key indication for emergent or urgent CABG.^5^ Although, prompt percutaneous coronary intervention evolved as the standard of care for STEMI. It is noteworthy that over the next 40+ years, most randomized trials of IMT versus coronary revascularization in CAD have reached similar conclusions except for the small subgroup of patients with LM stenosis or severe multiple vessel obstructive disease among patients with diabetes. Clearly, his work has changed the way we think about and approach patients with acute CAD.

Dick continued his contributions in acute and chronic CAD over the next several decades as a committee member for key national randomized trials of B‐Blockers in Myocardial Infarction, the Multiple Risk Factor Intervention Trial, the Thrombolysis in Myocardial Infarction trial, Chair of the NHLBI‐Asymptomatic Cardiac Ischemia Pilot multicenter, multinational study and the NHLBI Cardiology Advisory Committee.

Work that he termed “Provocative Pharmacoangiography”^6^ pioneered the use of “Functional Coronary Angiography” 40 years before its importance was recognized in the 2019 ESC Chronic IHD Guideline and the 2021 ACC/AHA Chest Pain Guideline.

## 4 CORONARY VASOMOTOR DISORDERS

Related to his close relationship with the Italian academic cardiologist Attilio Maseri, Dick was keenly interested in coronary artery spasm (CAS). He encouraged us to intensely study CAS as a key mechanism for ischemia, with or without obstructive atherosclerotic CAD.^7^ He encouraged us to follow such patients for their clinical outcomes. At one time, we had a registry that regularly followed over 500 patients with angiographically confirmed CAS. This included a series of studies in which we documented that spontaneous CAS and CAS provoked by intravenous ergonovine demonstrated by coronary angiography had essentially the same clinical and angiographic characteristics.^8^ We also used intracoronary ergonovine in an attempt to minimize its systemic vascular effects which elevated myocardial oxygen demands and confounded the actual cause of ischemia among patients with co‐existing obstructive CAD. Ergonovine was assessed in patients with mitral valve prolapse.^9^ We also studied the coronary artery responses to inhalation of cold air in the angiographic lab.^10^

We intensely investigated coronary angiographic responses to various medications. Nitroglycerin was studied intensively.^11^ These results led us to argue against the “routine use of nitroglycerin prior to coronary angiography” since its use would remove the possibility of documenting spontaneous CAS.^8^, ^11^, ^12^, ^13^, ^14^, ^15^, ^16^, ^17^ We studied most of the medications used for coronary spasm, often before they were approved for that indication.^18^, ^19^, ^20^, ^21^, ^22^

## 5 IMPORTANCE OF PHYSICAL CHARACTERISTICS OF CORONARY NARROWING

In the experimental physiology laboratory, Dick encouraged us to perform a series of studies to evaluate the functional importance of various physical characteristics of coronary artery narrowings in an animal model. We investigated the influence of the location of the stenosis, the length of the stenosis and multiple stenoses in series. This work helped to refine the understanding of these characteristics when observed in our patients.^23^, ^24^, ^25^, ^26^ We also examined the effects of various valvular lesions on the functional significance of coronary artery narrowings.^27^

## 6 CARDIOVASCULAR PHYSIOLOGY

When Dick moved to Florida, he also brought with him an R01 (HL 15576‐01 CVB, “Velocity of Aortic Blood Flow In Myocardial Ischemia”), and a cardiovascular physiologist, Wilmer Nichols, PhD. That program, Dr Nichols, and collaborations with Huntly Millar brought novel catheters with miniature micromanometers that provided high‐fidelity aortic and LV pressure and ascending aortic blood flow velocity, as well as pulmonary artery pressure and blood flow velocity.^28^ This work led to a series of publications about instantaneous force‐velocity‐length relations in the intact human heart and aortic input impedance as LV load.^28^, ^29^, ^30^, ^31^, ^32^, ^33^, ^34^ His R01 provided the resources to study aortic and pulmonary blood flow in intact patients: Our ability to perform aortic and pulmonary artery blood flow velocity measurements led to a series of studies in patents with myocardial ischemia and other disorders. We evaluated aortic input impendence in heart failure^30^, ^31^, ^32^, ^33^, ^34^ and also investigated pulmonary artery blood flow.^35^

## 7 CORONARY ANGIOPLASTY

As a former cardiac cath lab director at Johns Hopkins Hospital, he very interested in everything that we did in the lab and did cardiac catheterizations until the early 2000s. Dick was very interested in percutaneous coronary angioplasty after hearing Andreas Gruentzig present his animal studies and first patient. When I, as the UF cardiac cath lab director, traveled to Zurich to attend the mandatory demonstration course required by the catheter manufacturer, Dick accompanied me. He was fascinated that we could now work within the coronary arteries. This interest also led him to support George Abela, one of our prior fellows, to pioneer the study of Laser Angioplasty in occluded human atherosclerotic arteries and several animal models.^36^, ^37^, ^38^, ^39^, ^40^, ^41^, ^42^, ^43^

We also used novel sensors^28^, ^44^, ^45^ and were among the early investigators to study the use of intracardiac echocardiography.^46^

## 8 EDITORIAL ATTRIBUTES

Dick was a prolific writer who often reminded all of us that “it didn't happen if it's not written down.” He had over 700 scientific publications. His writings led to invitations to be Editor‐In‐Chief of the following: ACC Learning Center Highlights; the Adult Clinical Cardiology Self‐Assessment Program; ACC Extended Learning (ACCEL); ACCEL News; *Clinical Cardiology*; and *The Netter Collection of Medical Illustrations‐Cardiovascular System* 2nd Ed., Vol. 8., Elsevier Saunders. Also, he was an Editorial Board Member for essentially all the key cardiovascular journals at some time during his career.

In 1989, Dick worked with Chinese cardiologists and brought several of us to Beijing to kick off the inaugural scientific session of a meeting that became the Great Wall Congress. He returned to assist in the selection of topics and speak at this meeting each year thereafter until the COVID‐19 pandemic. In 2014, on the 25th anniversary of the Great Wall International Congress of Cardiology (GW‐ICC), Dick, along with Professor Dayi Hu, president of the GW‐ICC and past president of the Chinese Society of Cardiology, founded the journal *Cardiovascular Innovations and Applications*.

## 9 LEADERSHIP ROLE

Dick Conti led the CVD program at the UF College of Medicine through a period of tremendous growth in clinical cardiovascular medicine, research, and training. Under his leadership we became the group that cared for more patients, did more teaching and clinical research, than any other Division in the Department of Medicine, and more than most Departments in the College of Medicine. He served as Professor of Medicine, and Chief of the Division of Cardiology, with an adjunct appointment in the Department of Physiology, for a quarter of century.

During this period, he developed a division that achieved national and international recognition. We trained many who went on to lead cardiology programs at other institutions in the United States and abroad. Also, they have gone on to edit several CVD journals. For this work, he was awarded the newly created AHA Eminent Scholar Chair in Cardiovascular Education from the Palm Beach Chapter in 1988. After he retired, his work was recognized by the 2015 UF College of Medicine Faculty Council's Lifetime Achievement Award.

## 10 INTERNATIONAL COLLABORATIONS

Dick loved to travel and received the UF College of Medicine International Educator of the Year Award for contributions in education, service and research throughout Europe, Asia, Africa, Australia, South America, and the Soviet Union. He was a member of 10 cardiology professional societies outside of the United States and was an invited lecturer for multiple international cardiac societies. During his tenure as president of the ACC from 1989 to 1990, he was a key member who helped to foster a close relationship with the European Society of Cardiology by creating reciprocal international symposia, which persist today and have led to invaluable sharing of scientific information.

## 11 MENTORSHIP

Since starting at the UF in 1974, I estimate that CR Conti mentored more than 150 fellows in training and junior faculty. Many of these served as, or continue to serve, the Division's faculty. As a past president of the ACC, he continued his teaching and mentoring through clinical practice. His audio journal ACCEL features interviews with researchers on various cardiology topics. He has also served as chairman of the American College of Cardiology Extramural Program Committee. As chairman of the Self‐Study Educational Programs Committee, he led the development of the initial ACC Self‐Assessment Program.

With his down‐to‐earth nature and no‐nonsense attitude, Dick was committed to excellence in patient care and education. During his career, he held positions on most American College of Cardiology (ACC) Committees and Task Forces, including the Board of Trustees, and was elected President 1989–1990. During his long tenure (2000–2010) as Editor of the ACC's audio journal ACCEL, his commitment to education was well documented in the recordings of his probing interviews and commentaries. Dick worked effortlessly to encourage medical students, residents, and fellows to become involved in academic pursuits. He was a key part of the Florida ACC Chapter for over 3 decades and initiated the Chapter's Young Investigator activities.

## 12 IN CLOSING

Dick Conti was incredible in his persistence for excellence in patient care and education, and these traits were also reflected in all other aspects of his work. He passed this trait on to those who worked with him.

Simply put, he made us all better cardiologists and better people. The national and international cardiology community lost an important part of their history with Dick's passing.

## Data Availability

Not available.

## References

[1] Conti CR . Current status of randomized prospective study on unstable angina. Adv Cardiol. 1978;22:130‐137. 10.1159/000401023 619513

[2] Russell RO , Moraski RE , Kouchoukos N , et al. Unstable angina pectoris: national cooperative study group to compare medical and surgical therapy. I. Report of protocol and patient population. Am J Cardiol. 1976;37:896‐902. 1266755 10.1016/0002-9149(76)90116-8

[3] Russell RO , Moraski RE , Kouchoukos N , et al. Unstable angina pectoris: national cooperative study group to compare surgical and medical therapy. II. In‐hospital experience and initial follow‐up results in patients with one, two and three vessel disease. Am J Cardiol. 1978;42:839‐848. 309277 10.1016/0002-9149(78)90105-4

[4] Russell RO , Moraski RE , Kouchoukos N , et al. Unstable angina pectoris: national cooperative study group to compare surgical and medical therapy. III. Results in patients with S‐T segment elevation during pain. Am J Cardiol. 1980;45:819‐824. 6965816 10.1016/0002-9149(80)90127-7

[5] Russell RO , Moraski RE , Kouchoukos NT , et al. Unstable angina pectoris: national cooperative study group to compare medical and surgical therapy. IV. Results in patients with left descending coronary artery disease. Am J Cardiol. 1981;48:517‐524. 6973924 10.1016/0002-9149(81)90082-5

[6] Conti CR , Curry RC , Christie LG , Pepine CJ . Clinical use of provocative pharmacoangiography in patients with chest pain. Adv Cardiol. 1979;26:44‐54. 420061 10.1159/000402388

[7] Curry RC Jr. , Pepine CJ , Sabom MB , Feldman RL , Christie LG , Conti CR . Effects of ergonovine in patients with and without coronary artery disease. Circulation. 1977;56:803‐809. 912842 10.1161/01.cir.56.5.803

[8] Curry RC Jr. , Pepine CJ , Sabom MB , Conti CR . Similarities of ergonovine‐induced and spontaneous attacks of variant angina. Circulation. 1979;59:307‐312. 103658 10.1161/01.cir.59.2.307

[9] Sabom MB , Curry RC, Jr. , Pepine CJ , Christie LG , Conti CR . Ergonovine testing for coronary artery spasm in patients with angiographic mitral valve prolapse. Cathet Cardiovasc Diagn. 1978;4:265‐274. 737730 10.1002/ccd.1810040308

[10] Feldman RL , Whittle JL , Pepine CJ , Conti CR . Regional coronary angiographic observations during cold stimulation in patient with exertional chest pain: comparison of diameter responses in normal and fixed stenotic vessels. Am Heart J. 1981;102:822‐830. 7304392 10.1016/0002-8703(81)90031-4

[11] Feldman RL , Pepine CJ , Curry RC, Jr. , Conti CR . Case against routine use of glyceryl trinitrate before coronary angiography. Br Heart J. 1978;40:992‐997. 101222 10.1136/hrt.40.9.992 PMC483522

[12] Feldman RL , Curry RC, Jr. , Pepine CJ , Mehta J , Conti CR . Regional coronary hemodynamic effects of ergonovine in patients with and without variant angina. Circulation. 1980;62:149‐159. 7379277 10.1161/01.cir.62.1.149

[13] Pepine CJ , Feldman RL , Conti CR . Recommendations for use of ergonovine to provoke coronary artery spasm. Cathet Cardiovasc Diagn. 1980;6:423‐426. 6781755 10.1002/ccd.1810060411

[14] Feldman RL , Pepine CJ , Conti CR . Magnitude of dilatation of large and small coronary arteries of nitroglycerin. Circulation. 1981;64:324‐333. 6788401 10.1161/01.cir.64.2.324

[15] Pepine CJ , Feldman RL , Conti CR . Action of intracoronary nitroglycerin in refractory coronary artery spasm. Circulation. 1982;65:411‐414. 6797753 10.1161/01.cir.65.2.411

[16] Whittle JL , Feldman RL , Pepine CJ , Curry RC , Conti CR . Variability of electrocardiographic responses to repeated ergonovine provocation in variant angina patients with coronary artery spasm. Am Heart J. 1982;103:161‐167. 7055051 10.1016/0002-8703(82)90488-4

[17] Feldman RL , Hill JA , Whittle JL , Conti CR , Pepine CJ . Electrocardiographic changes with coronary artery spasm. Am Heart J. 1983;106:1288‐1297. 6650351 10.1016/0002-8703(83)90036-4

[18] Feldman RL , Pepine CJ , Whittle J , Conti CR . Short‐ and long‐term responses to diltiazem in patients with variant angina. Am J Cardiol. 1982;49:554‐559. 7036708 10.1016/s0002-9149(82)80011-8

[19] Hill JA , Feldman RL , Pepine CJ , Conti CR . Randomized double‐blind comparison of nifedipine and isosorbide dinitrate in patients with coronary arterial spasm. Am J Cardiol. 1982;49:431‐438. 6800251 10.1016/0002-9149(82)90521-5

[20] Mehta J , Pepine CJ , Day M , Guerrero JR , Conti CR . Short‐term efficacy of oral verapamil in rest angina. A double‐blind placebo controlled trial in CCU patients. Am J Med. 1981;71:977‐982. 7032291 10.1016/0002-9343(81)90323-5

[21] Pepine CJ , Feldman RL , Hill JA , et al. Clinical outcome after treatment of rest angina with calcium blockers: comparative experience during the initial year of therapy with diltiazem, nifedipine, and verapamil. Am Heart J. 1983;106:1341‐1347. 6359845 10.1016/0002-8703(83)90043-1

[22] Pepine CJ , Feldman RL , Whittle J , Curry RC , Conti CR . Effect of diltiazem in patients with variant angina: a randomized double‐blind trial. Am Heart J. 1981;101:719‐725. 6786067 10.1016/0002-8703(81)90606-2

[23] Conti CR , Pepine CJ , Feldman RL , Nichols WW . The angiographic definition of critical coronary stenosis. Acta Med Scand Suppl. 1978;615:9‐17. 99986 10.1111/j.0954-6820.1978.tb17493.x

[24] Feldman RL , Nichols WW , Pepine CJ , Conetta DA , Conti CR . The coronary hemodynamics of left main and branch coronary stenoses. The effects of reduction in stenosis diameter, stenosis length, and number of stenoses. J Thorac Cardiovasc Surg. 1979;77:377‐388. 762981

[25] Feldman RL , Nichols WW , Pepine CJ , Conti CR . Hemodynamic significance of the length of a coronary arterial narrowing. Am J Cardiol. 1978;41:865‐871. 645594 10.1016/0002-9149(78)90726-9

[26] Feldman RL , Nichols WW , Pepine CJ , Conti CR . Hemodynamic effects of long and multiple coronary arterial narrowings. Chest. 1978;74:280‐285. 688785 10.1378/chest.74.3.280

[27] Feldman RL , Nichols WW , Pepine CJ , Conti CR . Influence of aortic insufficiency on the hemodynamic significance of a coronary artery narrowing. Circulation. 1979;60:259‐268. 156092 10.1161/01.cir.60.2.259

[28] Nichols WW , Pepine CJ , Millar HD , Christie LG Jr. , Conti CR . Percutaneous left ventricular catheterisation with an ultraminiature catheter‐tip pressure transducer. Cardiovasc Res. 1978;12:566‐568. 737669 10.1093/cvr/12.9.566

[29] Nichols WW , Conti CR , Pepine CJ . Instantaneous force‐velocity‐length relations in the intact human heart. Am J Cardiol. 1977;40:754‐761. 920612 10.1016/0002-9149(77)90193-x

[30] Klinke WP , Christie LG , Nichols WW , et al. Use of catheter‐tip velocity—pressure transducer to evaluate left ventricular function in man: effects of intravenous propranolol. Circulation. 1980;61:946‐954. 7363437 10.1161/01.cir.61.5.946

[31] Nichols WW , O'rourke MF , Avolio AP , et al. Effects of age on ventricular‐vascular coupling. Am J Cardiol. 1985;55:1179‐1184. 3984897 10.1016/0002-9149(85)90659-9

[32] Nichols WW , Pepine CJ , Conti CR , Christie LG , Feldman RL . Evaluation of a new catheter‐mounted electromagnetic velocity sensor during cardiac catheterization. Cathet Cardiovasc Diagn. 1980;6:97‐113. 6988082 10.1002/ccd.1810060114

[33] Pepine CJ , Nichols WW , Conti CR . Aortic input impedance in heart failure. Circulation. 1978;58:460‐465. 679436 10.1161/01.cir.58.3.460

[34] Pepine CJ , Nichols WW , Curry RC Jr. , Conti CR . Aortic input impedance during nitroprusside infusion. A reconsideration of afterload reduction and beneficial action. J Clin Invest. 1979;64:643‐654. 457874 10.1172/JCI109505 PMC372162

[35] DeLoskey AF , Nichols WW , Conti CR , Pepine CJ . Estimation of beat‐to‐beat stroke volume from the pulmonary arterial pressure contour in man. Med Biol Eng Comput. 1978;16:707‐714. 310932 10.1007/BF02442451

[36] Abela GS , Conti CR , Normann S , Feldman RL , Pepine CJ . A new model for investigation of transluminal recanalization: human atherosclerotic coronary artery xenografts. Am J Cardiol. 1984;54:200‐205. 6741814 10.1016/0002-9149(84)90329-1

[37] Abela GS , Crea F , Seeger JM , et al. The healing process in normal canine arteries and in atherosclerotic monkey arteries after transluminal laser irradiation. Am J Cardiol. 1985;56:983‐988. 4072933 10.1016/0002-9149(85)90417-5

[38] Abela GS , Crea F , Smith W , Pepine CJ , Conti CR . In vitro effects of argon laser radiation on blood: quantitative and morphologic analysis. J Am Coll Cardiol. 1985;5:231‐237. 3968308 10.1016/s0735-1097(85)80042-5

[39] Abela GS , Normann S , Cohen D , Feldman RL , Geiser EA , Conti CR . Effects of carbon dioxide, Nd‐YAG, and argon laser radiation on coronary atheromatous plaques. Am J Cardiol. 1982;50:1199‐1205. 6816057 10.1016/0002-9149(82)90448-9

[40] Abela GS , Normann SJ , Cohen DM , et al. Laser recanalization of occluded atherosclerotic arteries in vivo and in vitro. Circulation. 1985;71:403‐411. 3965181 10.1161/01.cir.71.2.403

[41] Abela GS , Seeger JM , Barbieri E , et al. Laser angioplasty with angioscopic guidance in humans. J Am Coll Cardiol. 1986;8:184‐192. 3711515 10.1016/s0735-1097(86)80111-5

[42] Abela GS , Staples ED , Conti CR , et al. Immediate and long‐term effects of laser radiation on the arterial wall: light and electron microscope observation. Surg Forum. 1983;34:454‐456.

[43] Crea F , Abela GS , Fenech A , Smith W , Pepine CJ , Conti CR . Transluminal laser irradiation of coronary arteries in live dogs: an angiographic and morphologic study of acute effects. Am J Cardiol. 1986;57:171‐174. 3942064 10.1016/0002-9149(86)90974-4

[44] Nichols WW , O'Rourke MF , Avolio AP , Yaginuma T , Pepine CJ , Conti CR . Ventricular/vascular interaction in patients with mild systemic hypertension and normal peripheral resistance. Circulation. 1986;74:455‐462. 3742748 10.1161/01.cir.74.3.455

[45] Nichols WW , Pepine CJ , Geiser EA , Conti CR . Vascular load defined by the aortic input impedance spectrum. Fed Proc. 1980;39:196‐201. 7353677

[46] Conetta DA , Christie LG Jr. , Pepine CJ , Nichols WW , Conti CR . Intracardiac M‐mode echocardiography for continuous left ventricular monitoring: method and potential application. Cathet Cardiovasc Diagn. 1979;5:135‐143. 487417 10.1002/ccd.1810050207

